# Analysis of trauma admission data at an urban hospital in Maputo, Mozambique

**DOI:** 10.1186/s12245-016-0105-8

**Published:** 2016-02-19

**Authors:** Cátia Luciana Abdulfattáhe Taibo, Troy D. Moon, Orvalho A. Joaquim, Carlos R. Machado, Amina Merchant, Kelly McQueen, Mohsin Sidat, Elena Folgosa

**Affiliations:** Injury Prevention and Safety Promotion Research Unit, University of Eduardo Mondlane, Salvador Allende Avenue, 702 Maputo, Mozambique; Vanderbilt University Medical Center, Vanderbilt Institute for Global Health, 2525 West End Ave Suite 750, Nashville, TN 37203 USA; Faculty of Medicine, University of Eduardo Mondlane, Salvador Allende Avenue, 702 Maputo, Mozambique; Department of Surgery, University of Eduardo Mondlane, Salvador Allende Avenue, 702 Maputo, Mozambique; Division of Trauma and Surgical Critical Care, Vanderbilt University Medical Center, 12ll S. 21st Ave, 404 MAB, Nashville, TN 37212 USA; Department of Anesthesiology, Vanderbilt University Medical Center, 1301 Medical Center Drive, #4648 TVC, Nashville, TN 37232 USA

**Keywords:** Trauma, Injury, Sub-Saharan Africa, Mozambique

## Abstract

**Background:**

Trauma is a major public health concern. Worldwide, injuries resulted in 4.8 million deaths in 2013, an increase of 11 % since 1990. The majority of deaths from trauma in low-and middle-income countries occur in a pre-hospital setting. Morbidity from trauma contributes significantly to disability in these countries. Mozambique has experienced a rise in injury-related morbidity and mortality. Efforts are underway to prioritize surgical and anesthesiology care in the post-2015 Global Surgery agenda that will build on momentum of the Millennium Development Goals. Injury surveillance remains vital to defining priorities and implementing policy changes.

**Methods:**

We performed a cross-sectional study between June and September, 2010 at the Hospital Central de Maputo (HCM). Data were collected on all patients admitted to the HCM emergency surgical services with a diagnosis of trauma. We describe patient characteristics and mechanism of traumatic injury by calculating simple proportions (for dichotomous or categorical variables) or medians with interquartile ranges (IQR) for continuous variables. Multivariable logistic regression analysis was used to estimate the mechanisms of trauma most associated with alcohol consumption.

**Results:**

A total of 517 patients were approached for inclusion in this study. Of these, 441 (91.5 %) participants were followed from admission until discharge. Three hundred twenty-four participants (73.5 %) were male. The most common age group was 20–29 years old. The three principal mechanisms of injury were road traffic injury, fighting, and falls, accounting for 74 % of injuries recorded. Traumatic injury involving alcohol consumption was nine times more likely to occur at a recreation/sporting event (OR 9.0, 95 % CI 3.01–27.13, *p* ≤ 0.0001).

**Conclusions:**

As Mozambique prepares to respond to the post-2015 international development agenda, urgent action is required to scale-up its national injury surveillance networks. Injury prevention efforts in Mozambique should focus attention on improving road safety regulations and their implementation, as well as on interventions targeting violence reduction and the reduction of alcohol consumption at sporting events.

## Background

Worldwide, injuries resulted in 4.8 million deaths in 2013, an increase of 11.0 % since 1990 [1]. Most of these deaths occurred in low- and middle-income countries (LMIC), with the World Health Organization (WHO) estimating that injury is responsible for more deaths than HIV, malaria, and tuberculosis combined [2–4]. The cost of trauma related injury remains exorbitant, both in terms of the economic consequences for society, as well as the fact that they account for 11 % of estimated disability adjusted life years (DALYS) for those living with the sequela of injury [5, 6]. These sequelae disproportionately affect LMIC due to inadequate surgical and anesthesiology services, poor rehabilitation systems, and minimal social safety nets [2, 7].

It is estimated, that of patients that die of trauma related injuries in LMIC, roughly 80.0 % die in the pre-hospital setting [5, 8]. Reductions in injury-associated morbidity and mortality have been linked to a variety of factors including adequate pre-hospital care, decreased transportation time to definitive care, reinforced transportation laws to diminish road traffic injuries (RTI), and improved injury surveillance [5, 6, 9]. Moreover, the addition of basic surgical services could avert approximately 21.0 % of the injury burden of LMIC [10]. As such, new attention and efforts are being made to prioritize both essential surgical and anesthesiology care in the post-2015 international agenda that will build on momentum and successes of the Millennium Development Goals (MDG) [7, 10, 11].

Mozambique is a resource-limited country on the southeast coast of Africa with a population of 25 million persons [5]. It ranked 178 of 187 nations on the 2014 United Nations Development Program (UNDP) Human Development Index (HDI), with a gross national income estimated at US $1011 per capita [12]. Mozambique’s health expenditure has risen substantially over the past 10 years, though as a proportion of total gross domestic product (GDP) it was only 6.8 % in 2013 ($71 USD per capita) [13]. In 2011, the total number of physicians registered in the National Health system was 1268, of whom 23.0 % were non-Mozambican nationals [14].

**Fig. 1:**
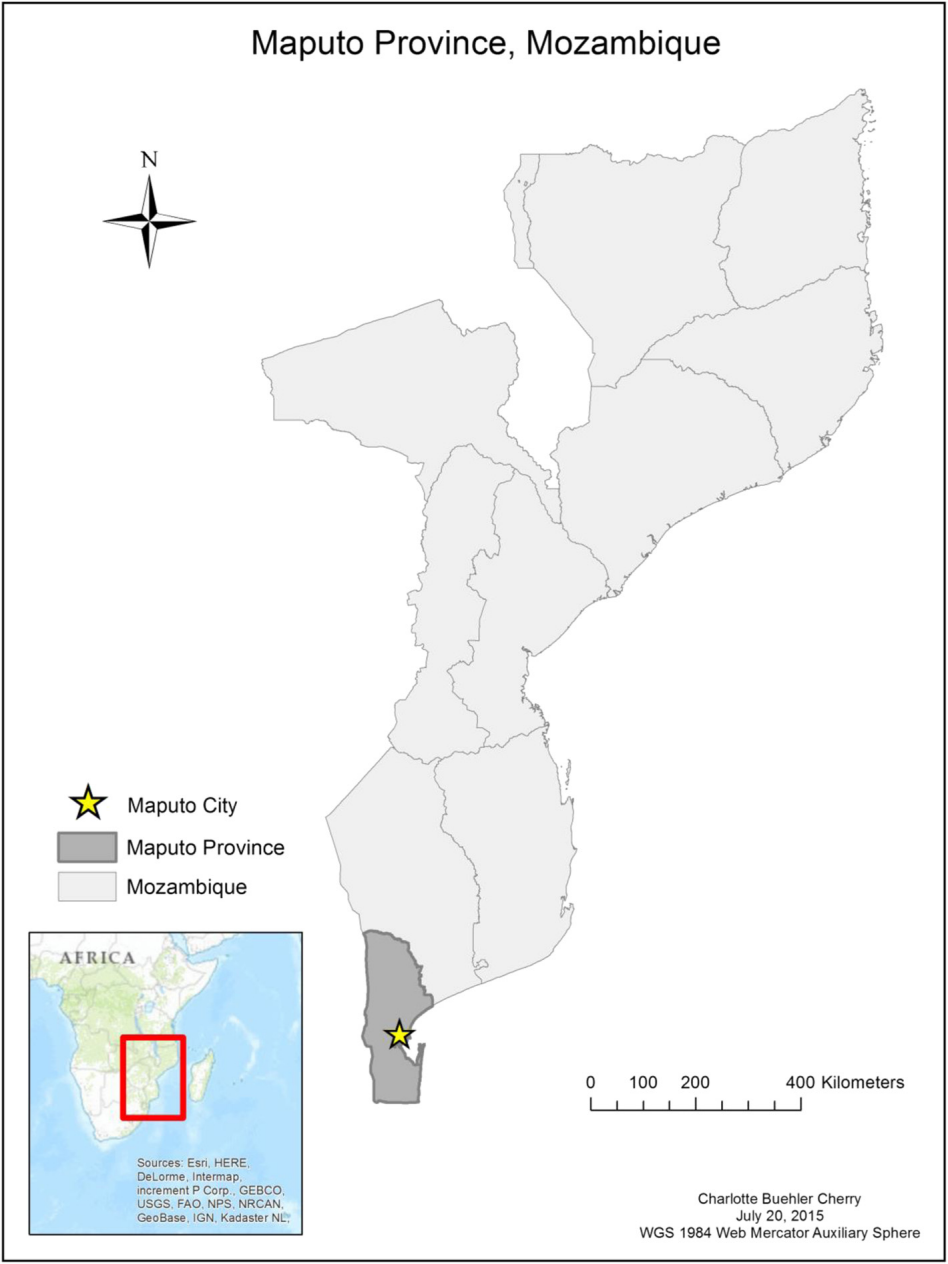
Map of Mozambique with Maputo City highlighted. Map created by Charlotte Buehler Cherry, Vanderbilt Institute for Global Health, July 23, 2015, Map Projection WGS 1984 Web Mercator Auxiliary Sphere, ArcGIS 10.2

*Hospital Central de Maputo* (HCM) is a 1500-bed quaternary care national reference center located in the capital city of Mozambique, Maputo (Fig. 1). It is the cornerstone hospital of the Mozambique National Health System and serves a population of approximately three million persons within Maputo and its surrounding catchment areas. It is the only public hospital in the country equipped to handle high-level advanced surgery, thereby serving as a principal referral center for the entire country [15].

Trauma-related morbidity and mortality is a growing concern. In its 2001–2005 health sector strategic plan, the Ministry of Health of Mozambique declared that trauma had reached “epidemic proportions” [16]. Increased motorization has contributed to a rapid rise of RTI in recent years. Pedestrians alone accounted for 55.0 % of road traffic deaths between 1993 and 2000 [17, 18]. In Maputo amongst all registered deaths in 2000, RTI were most common (43.7 %), followed by firearms (8.7 %), and burns (7.8 %) [19]. In contrast, for children aged 0–14 in 2007 in Maputo, falls (40.6 %) were the most common mechanism of injury, followed by burns (19.1 %), and RTI (14.3 %) [16]. In 2001, the analysis of Mozambican national mortality data revealed that trauma was the second leading cause of death in persons aged 15–59 years and amongst the top 10 causes of death for all age groups [18].

## Methods

We performed a cross-sectional study between June and September, 2010 at the HCM, utilizing a standardized trauma registry specifically created for this study. Data were collected on all patients admitted to the HCM emergency services meeting the following eligibility criteria: ≥18 years of age and an admission diagnosis of physical trauma that occurred within the previous 24 h. Following the collection of informed consent, data were collected on demographics, mechanism of injury, location where injury occurred, and consumption of alcohol. Patients were followed by study personnel until the date of discharge from the hospital.

Data were entered into EpiData version 3.0 (Lauritsen JM. (Ed.) EpiData Data Entry, Data Management and Basic Statistical Analysis System. Odense Denmark, EpiData Association, 2000–2008. http://www.epidata.dk) and then transferred to Stata version 11 (StataCorp. 2009. Stata: Release 11. Statistical Software. College Station, TX: StataCorp LP) for data analysis.

We describe participant characteristics by calculating simple proportions (for dichotomous or categorical variables) or medians with interquartile ranges (IQR) for continuous variables. Pearson’s chi-square or Fisher’s exact test was used to compare the distribution of one categorical variable amongst levels of the other variables. To estimate the odds ratio, logistic regressions models were built with minimal adjustment for age and gender.

The study was approved by the Mozambique National Bioethics Committee for Health (*Comité Nacional de Bioética em Saúde*, CNBS).

## Results

A total of 517 patients were approached for inclusion in this study. Of these, 35 (6.8 %) refused to participate. Of the 482 patients who accepted, we were able to collect data and follow 441 (91.5 %) participants from admission to discharge from the hospital and the study. Reasons identified for loss of follow-up included transfer to another facility (1.5 %) and inappropriate or poor documentation making it impossible to find the patient discharge paperwork (7.0 %).

Of the 441 patients included in the analysis, 324 (73.5 %) were male. Participant ages ranged from 18–90 years old, with the median age of all participants being 28 (IQR 23–37). For both men and women admitted for trauma, the most common age group was 20–29 years old. The most common marital status was “single” (53.1 %), followed by “married” (40.8 %). Close to 50.0 % of women were domestic workers, while the most common occupations for men were private sector employee (23.1 %), day laborer (20.1 %), student (16.7 %), unemployed (12.0 %), and public servant (11.4 %) (Table 1).

**Table 1:** Socio-demographic characteristics of adults admitted to Hospital Central de Maputo for trauma related injuries, June–September, 2010

	Male	Female	Total	*P* value
	*n* = 324 (%)	*n* = 117 (%)	*n* = 441 (%)	
Age^a^	28 (23–35)	29 (24–44)	28 (23–37)	0.09
18–19	13.6	12.8	13.4	0.008
20–29	40.7	38.5	40.1	
30–39	27.8	19.7	25.6	
40–49	10.8	10.3	10.7	
>50	7.1	18.8	10.2	
Marital status				<0.0001
Single	56.2	44.4	53.1	
Married	39.5	44.4	40.8	
Divorced	0.6	2.6	1.1	
Widowed	0.9	5.1	2.0	
No response	2.8	3.4	2.9	
Occupation				<0.001
Student	16.7	13.7	15.9	
Domestic worker	3.7	48.7	15.6	
Public servant	11.4	7.7	10.4	
Private sector employee	23.1	9.4	19.5	
Day laborer	20.1	6.0	16.3	
Business owner	1.5	1.7	1.6	
Agriculture	1.9	0.0	1.4	
Retired	0.9	1.7	1.1	
Unemployed	12.0	4.3	10.0	
No response	8.6	6.8	8.2	

^a^Continuous variables are listed as median (interquartile range)

The three principal mechanisms of injury for patients admitted with trauma were RTI, fighting (defined as an incident with someone outside of their domestic unit), and falls (Table 2). Together, these three accounted for 74.0 % of injuries recorded. RTI were reported for 173 (39.2 %) participants, with a statistically significant higher proportion of cases happening amongst women. The majority of traffic-related injuries (57.0 %) were caused as a result of a pedestrian being struck by a motor vehicle. Females involved in motor vehicle accidents were more likely to be a passenger (40.0 %) than the driver (4.0 %) of the vehicle.

**Table 2:** Mechanism of injury for patients admitted to Hospital Central de Maputo with trauma related injuries, June–September, 2010

	Male	Female	Total	*P* value
	*n* = 324 (%)	*n* = 117 (%)	*n* = 441 (%)	
Road traffic injury	116 (35.8)	57 (48.7)	173 (39.2)	0.014
Pedestrian	68 (59.0)	32 (56.0)	100 (57.8)	
Bicyclist	2 (2.0)	0 (0.0)	2 (1.0)	
Vehicle passenger^a^	29 (25.0)	23 (40.0)	52 (30.0)	
Car driver	12 (10.0)	2 (4.0)	14 (8.0)	
Motorcyclist	5 (4.0)	0 (0.0)	5 (3.0)	
Fall	31 (9.6)	10 (8.5)	41 (9.3)	0.744
From a height (building, stairs, etc.)	15 (48.0)	2 (20.0)	17 (41.0)	
From a tree	5 (16.0)	1 (10.0)	6 (15.0)	
From ground level/back of vehicle	8 (26.0)	7 (70.0)	15 (37.0)	
Burn	17 (5.2)	19 (16.2)	36 (8.2)	<0.001
Hot liquid	7 (41.0)	6 (32.0)	13 (36.0)	
Fire	9 (53.0)	9 (47.0)	18 (50.0)	
Chemical product	0 (0.0)	1 (5.0)	1 (3.0)	
Other	1 (6.0)	3 (16.9)	4 (11.0)	
Fighting	104 (32.1)	8 (6.8)	112 (25.4)	<0.001
Knife wound	25 (7.7)	4 (3.4)	29 (6.6)	0.108
Gun wound	16 (4.9)	1 (0.9)	17 (3.9)	0.049
Domestic violence	1 (0.3)	9 (7.7)	10 (2.3)	<0.001
Work related accident	5 (1.5)	1 (0.9)	6 (1.4)	0.582
Bites	8 (2.5)	7 (6.0)	15 (3.4)	0.072
Human	6 (75.0)	4 (57.0)	10 (66.0)	
Snake	1 (13.0)	2 (29.0)	3 (20.0)	
Other animal	1 (13.0)	1 (14.0)	2 (14.0)	

^a^Includes passengers of public and semi-public transport

Overall, the second most common mechanism of traumatic injury resulted from fighting (25.4 %), though significant differences were seen amongst the sexes. Fighting represented 32.1 % of injuries reported by males vs. 6.8 % of female injuries. In contrast, 7.7 % of female injuries resulted from reported domestic violence, while domestic violence represented less than 1.0 % of male injuries.

**Fig. 2:**
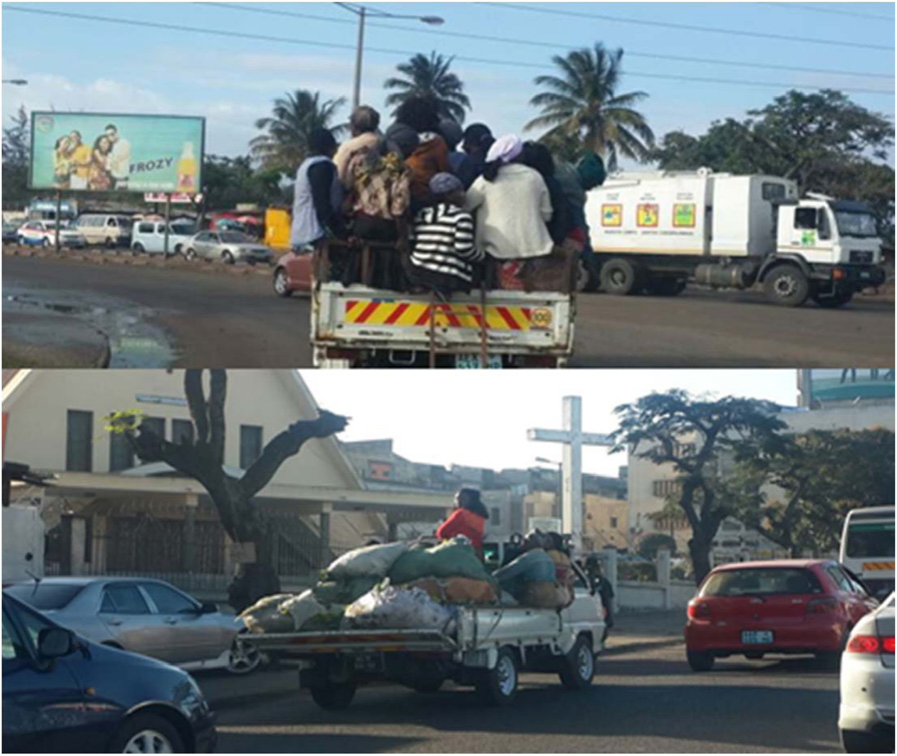
“My Love” semi-public transport vehicle

Falls were reported as the cause of trauma in 41/441 (9.3 %) cases. Most falls occurred in males (31/41, 76 %), with 48.0 % occurring from some height, 26.0 % from ground level or from the back of a semi-public transport vehicle (Fig. 2), and 16.0 % from trees. Falls from the back of a semi-public transport vehicle are a common occurrence in Maputo, most often resulting from too many passengers and not a collision with another vehicle for example. As such, these injuries are not classified as RTI on HCM admission paperwork.

Data on alcohol consumption were available for 142/441 (32.2 %) cases of trauma. Evidence of alcohol consumption was seen in 55/173 (32.0 %) of RTI cases, 44/112 (39.0 %) cases of fighting, and 10/41 (24.0 %) of falls. In logistic regression models of analysis, the mechanisms of trauma most associated with alcohol consumption were knife wound (OR 2.6, 95 % CI 1.12–5.90, *p* = 0.02) and fighting (OR 1.9, 95 % CI 1.16–3.05, *p* = 0.015). In contrast, persons admitted due to burns were 80.0 % less likely to have consumed alcohol (OR 0.2, 95 % CI 0.07–0.60, *p* = 0.003) (Table 3). The top three locations for alcohol-related injuries were public spaces (83/142 [58.5 %]), sites of recreation/sports (24/142 [16.9 %]), and at home (22/142 [15.5 %]). In logistic regression models, traumatic injury involving alcohol consumption was nine times more likely to occur a recreation/sporting event (OR 9.0, 95 % CI 3.01–27.13, *p* = <0.0001).

**Table 3:** Logistic regression for mechanism of injury and consumption of alcohol for patients admitted to Hospital Central de Maputo with trauma related injuries, June–September, 2010

	OR	(95 % CI)	*P* value
Road traffic injury	1.0	(0.68–1.61)	0.932
Pedestrian	1.3	(0.82–2.21)	0.300
Car passenger	0.5	(0.23–1.02)	0.075
Car driver	2.7	(0.85–8.31)	0.145
Motorcyclist	0.5	(0.06–5.16)	0.971
Fall	0.5	(0.25–1.15)	0.150
From a height (building, stairs, etc.)	0.5	(0.15–1.50)	0.301
From a tree	0.3	(0.04–2.74)	0.497
From ground level/back of vehicle	0.8	(0.24–2.70)	0.949
Burn	0.2	(0.07–0.60)	0.003
Hot liquid	0.2	(0.02–1.38)	0.123
Fire	0.2	(0.05–0.90)	0.040
Other	0.2	(0.07–0.60)	0.003
Fighting	1.9	(1.16–3.05)	0.015
Knife wound	2.6	(1.12–5.90)	0.020
Gun wound	1.2	(0.41–3.57)	0.943
Work-related accident	0.4	(0.04–3.59)	0.698
Bites	1.1	(0.37–3.08)	0.889
Human	1.6	(0.46–5.73)	0.662
Snake	0.8	(0.07–8.92)	0.966

Data on the anatomic site of injury showed that traumatic brain injuries (TBIs) were the most common overall (Table 4). For those reporting RTI, 73.4 % (127/173) presented with TBI, 36.6 % (53/173) with a maxillofacial injury, 23.7 % (41/173) with external contusions, and 22.0 % (38/173) with an orthopedic injury. For persons reporting “falls” as the cause of injury, 61.0 % (25/41) were TBI, 19.5 % (8/41) were cervical spine, and 12.2 % (5/41) were thoracic. For persons reporting “fighting” as the cause of injury, 66.1 % (74/112) were TBI, 46.4 % (52/112) were maxillofacial, and 11.6 % (13/112) were external contusions. The principal site for knife wound injuries was thoracic (44.8 %) and gun wound injuries was abdominal (47.1 %).

**Table 4:** Anatomic site of injury by mechanism of trauma

	Number (%)
Road traffic injury (*n* = 173)	
Cranioencephalic	127 (73.4)
Maxillofacial	53 (36.6)
External contusion	41 (23.7)
Orthopedic	38 (22.0)
Fall (*n* = 41)	
Cranioencephalic	25 (61.0)
Cervical spine	8 (19.5)
Thoracic	5 (12.2)
Fighting (*n* = 112)	
Cranioencephalic	74 (66.1)
Maxillofacial	52 (46.4)
External contusion	13 (11.6)
Knife wound (*n* = 29)	
Thoracic	13 (44.8)
Cranioencephalic	8 (27.6)
Gun wound (*n* = 17)	
Abdominal	8 (47.1)
Thoracic	4 (23.5)

## Discussion

Mozambique currently has no active national trauma surveillance system. As such, this study provides important insights into the patterns of injury seen upon admission into the principal hospital of the country’s largest reference center. We show that the population most affected by traumatic injury was young, non-married, male adults between the ages of 20–29 years and that most injuries occurred as a result of road traffic injury, violence-related injuries such as fighting, and falls.

Developed countries have seen steady reductions in traffic-related injuries in the last several decades, almost all resulting from changes in road use behavior, improvements in vehicle safety standards, and strict enforcement of traffic safety laws [20, 21]. Meanwhile, RTI continue to plague LMIC, accounting for a sizeable proportion of DALYS lost [8, 22]. Mozambique, like other countries, is no different and over the last 25 years has experienced a significant increase in the number of private-owned vehicles in circulation, as well as an insufficient and often overcrowded/unsafe public transport system. According to government records, the number of registered private vehicles in Mozambique increased from 52,239 in 1990 to 542,336 in 2013, with approximately 75.0 % of these being in Maputo [23]. In our study, we found that 57.8 % of RTI occurred in pedestrians and another 30.0 % occurred amongst vehicle passengers. This finding is consistent with other LMIC studies which found that most RTI occurred in those most vulnerable and least protected, being pedestrians, unrestrained passengers, bicyclists, and motorcyclists [24]. Females in our study involved in RTI reported a higher proportion of traumatic injuries resulting while being a vehicle passenger. Many Mozambican women, in the southern region in particular, contribute to their household economics by selling agricultural products at informal shops and markets. In a majority of cases, the only transport options available for taking their products to market are the large fleets of private-owned, small mini-buses called “Chapas” or open-backed trucks nicknamed “My Love”, which offer little or no protection in the case of an accident.

Violent injuries from “fighting” were the reported cause in 25.0 % of traumatic injuries requiring admission to HCM. While this accounted for a substantial proportion of injuries seen, the number of violent injuries resulting from either a knife or gunshot wound was considerably smaller (6.6 and 3.9 %, respectively). Mozambique is not a country at war and is not typically considered to have a problem with violence such as its neighboring country South Africa. In South Africa for example, the second leading cause of mortality in 2000 was “violence”-related injuries and occurred most frequently amongst males aged 15–29 years old [25]. While not as widespread, violence in Mozambique also appears to disproportionally affect young males and is strongly associated with the consumption of alcohol. We found that fighting and knife wounds as the cause of injury on hospital admission were twice as likely when alcohol was involved (OR 1.9; 95 % CI = 1.16–3.05, *p* = 0.015 and OR 2.6; 95 % CI = 1.12–5.90, *p* = 0.02, respectively). Of particular interest is the strong association found between the consumption of alcohol at a recreational/sporting event. This finding is consistent with WHO findings that alcohol-related traumatic injuries are more likely to occur in public places and during periods of leisure and play [26].

Falls were the third most common form of injury seen in our study. This likely reflects high-risk activities such as the use of open-backed trucks for public transportation and harvesting practices in rural areas consisting of climbing trees for coconuts and other fruits.

Establishing a formalized trauma registry with financial support will shed light on trauma background, severity, and risk factors for morbidity and mortality. An established registry will also allow Mozambique, as well as other developing countries, to understand the causes of trauma and focus prevention efforts and financial commitments to high-impact solutions. In order to develop policy, the basic information in a trauma registry must be collected and evaluated on an ongoing, consistent basis. Changes can be directly based on verified data. A trauma registry will also highlight what types of pre-hospital care must be provided. Currently, no formal public ambulance system exists, and no universal emergency number is functional. When trauma occurs, Mozambicans are reliant on private assistance if they can afford it or public goodwill. In LMIC, pre-hospital care is vital to survival as most deaths occur within the first few hours of injury. In many low-income countries, trained health workers and police played a negligible role in the provision of first aid and on-scene treatment of road traffic injuries (RTI) [27]. Given the financial and resource constraints in low-income countries, simple but systematic pre-hospital training programs have been implemented in rural villages to stabilize patients. Most pre-hospital deaths are the result of airway compromise, respiratory failure or uncontrolled hemorrhage; all three of these conditions can be addressed using basic first aid measures [5]. Supporting this information with data from a trauma registry can improve efforts toward pre-hospital care.

Once a patient is admitted to the hospital after a trauma, appropriate services must be available. Trauma requires integrated services from multiple specialties including neurosurgery, orthopedic surgery, anesthesiology, general surgery, and otolaryngology in addition to adequate blood products, surgical tools, and intensive care. A comprehensive trauma registry that follows patients from admission to discharge and beyond, will characterize necessary resources and current assets, along with areas of improvement for trauma care. This information allows policy adjustments and resource allocation based on verified, dynamic data.

## Conclusions

Traumatic injuries contribute significantly to the burden of disease in Mozambique, especially in the capital city Maputo. This study likely underestimates the true prevalence of traumatic injuries in Maputo as it represents only those injuries attended to in a hospital setting. Like many sub-Saharan countries, the paucity of surveillance data in Mozambique leads to uncertainties, though information represented here is consistent with studies in other similar LMIC settings. Injury prevention efforts in Mozambique should focus attention on improving road safety regulations and their implementation, as well as on interventions targeting violence reduction and the reduction of alcohol consumption at sporting events.

As Mozambique prepares to respond to the post-2015 international development agenda, urgent action is required to scale-up its national injury surveillance networks in order to inform policy makers as to needed priorities for improved prevention activities and improved access to both emergency and trauma care.
